# Family Socioeconomic Status and Exposure to Childhood Trauma: Racial Differences

**DOI:** 10.3390/children7060057

**Published:** 2020-06-03

**Authors:** Shervin Assari

**Affiliations:** Department of Family Medicine, Charles R Drew University of Medicine and Science, Los Angeles, CA 90059, USA; assari@umich.edu; Tel.: +1-734-232-0445; Fax: +734-615-8739

**Keywords:** race, ethnicity, socioeconomic status, trauma, stress, stressful life events

## Abstract

Background: Minorities’ diminished returns (MDRs) refer to weaker effects of socioeconomic status (SES) indicators such as parental educational attainment and family income in generating tangible childhood outcomes for racial and ethnic minorities compared to the majority group, a pattern prevalent in the US. Our existing knowledge is minimal, however, about diminished returns of family SES on reducing exposure to childhood trauma. Aim: To determine if there was a difference between non-Hispanic whites (NHW) and non-Hispanic blacks (NHB) in the effect of SES on exposure to childhood trauma among children ages 8–11 years old. Materials and methods: In this cross-sectional study, we analyzed data from 4696 NHW or NHB American 8–11-year-old children who were participants in the Adolescent Brain Cognitive Development (ABCD) Study. The independent variables were parental educational attainment and family income. The primary outcome was exposure to 1 or 2+ childhood traumas, measured by the Kiddie Schedule for Affective Disorders and Schizophrenia (K-SADS) semi-structured interview. Polynomial regression was used for data analysis. Results: Parental education and family income had statistically significant protective (negative) effects on childhood trauma, indicating children from high income and highly educated families were exposed to a lower level of childhood trauma. However, race/ethnicity showed statistically significant interactions with parental education and family income on exposure to childhood trauma, indicating weaker protective effects of parental education and family income on reducing exposure to trauma for NHB compared to NHW children. Race-specific models showed protective effects of parental education and family income on exposure to childhood trauma for NHW but not NHB children. Conclusion: The protective effects of parental education and family income against exposure to childhood trauma are systematically diminished for NHBs compared to NHWs. To minimize the racial/ethnic health gaps, diminished returns of parental education and family income should be addressed. There is a need for programs and interventions that equalize not only SES but also the marginal returns of SES for ethnic groups. Such efforts require addressing structural and societal barriers that hinder NHB families from translating their SES resources into tangible outcomes. There is a need for studies that can minimize MDRs for NHB families, such that SES can similarly secure tangible outcomes in the presence of SES resources.

## 1. Introduction

Adverse childhood experiences (ACEs), childhood trauma, or childhood stress are among the leading social determinants of health later in life [1]. Groups and individuals who experience higher levels of ACE, childhood trauma, adversities, or stress have worse health statuses in their adulthood. These effects are not specific and influence mental, physical, and physiological effects [1,2,3,4,5,6]. Some examples of the undesired effects of exposure to childhood trauma include anxiety [7], PTSD [8], depression [7], suicide [1,6], substance use [9,10,11], drug use [12], and mortality [1,2,3,4,5,6]. Childhood trauma also increases the risk of respiratory, heart, and metabolic diseases [13]. Childhood trauma deteriorates health through various mechanisms such as systemic inflammation [14], reducing telomere length [15,16,17,18], and DNA methylation [19,20,21]. Exposure to childhood trauma also impacts the developing brain [22].

Race [23] and socioeconomic status (SES) [24] closely correlate with each other as well as with exposure to childhood trauma [25]. Families with low SES report high levels of exposure to childhood trauma [25]. As stress is believed to be one of the mechanisms that connect SES to health [26], low exposure to childhood trauma may explain why high-SES individuals report better health [25]. One study showed that exposure to childhood trauma might mediate the effect of SES on health [27]. However, SES may differently reduce stress and trauma for non-Hispanic white (NHW) and non-Hispanic black (NHB) families [28,29,30,31] and children [32,33]. In one study, highly educated NHBs remained at a higher risk of poverty than highly educated NHWs [34,35]. In another study, NHBs reported high stress at all levels of social mobility; however, for NHWs, stress was a function of social mobility [36]. Several other studies have also shown that high-SES NHBs report very high levels of race-related stress and discrimination [28,29,30,31]. In other studies, highly educated NHBs experience a higher level of occupational stress, while highly educated NHWs reported the lowest level of occupational stress [37].

Recent evidence suggests that, relative to NHWs, NHB children show weaker effects of family income and other SES indicators such as parental education and marital status on tangible childhood and adulthood outcomes, also known as minorities’ diminished returns (MDRs) [38,39]. As shown by the MDR literature, various SES indicators, such as their own income and education [40] and that of their parents [35,41,42], may generate unequal outcomes for the members of diverse ethnic groups. Ethnic minority groups may differ in their ability to navigate resource systems for their resources to secure tangible outcomes in the presence of high educational attainment [39,42,43,44,45,46]. For example, NHB children show weaker effects of family income and parental educational attainment on various outcomes relative to their NHW counterparts [38,39,44,47,48]. We propose that the differential effects of family SES on exposure to childhood trauma may play a role in explaining MDRs.

Although there are very few, if any, studies on MDRs of family SES on exposure to childhood trauma, there are several reasons for such an expectation. First, although MDRs of parental SES on youth outcomes are shown, the role of exposure to childhood stress is not known in this regard. One can say that a reason youth and children from highly educated and high-income families still report a high level of depression and anxiety and aggression is a high level of stress and trauma in their life. Second, there is some evidence suggesting that highly educated families may experience a higher level of stress and trauma. It has been shown that an increase in SES does not decrease, but rather increases their exposure to discriminatory stressful experiences. Similarly, high parental education does not similarly enhance the work conditions of non-white families. Finally, non-white families with high SES are more likely to be surrounded by white individuals, which increases their discrimination.

### Aims

To determine if there was a difference between NHB and NHW children for the effects of parental educational attainment and family income on exposure to childhood trauma. We expected weaker effects of parental educational attainment and family income on exposure to childhood trauma for NHB than NHW children. Full support of our hypothesis would propose that exposure to childhood trauma in high-SES NHB families may be why high-SES NHB youth remained at increased risk of depression anxiety, and aggression (i.e., MDRs) [38,39].

## 2. Methods

### 2.1. Design and Settings

This was a secondary analysis of the Adolescent Brain Cognitive Development (ABCD) study [49,50,51,52,53]. This was a cross-sectional analysis of the ABCD data. ABCD is a national, state-of-the-art brain imaging study of childhood brain development. More information about ABCD’s purpose, methodology, and measurement is available elsewhere [49,54].

### 2.2. Advantage of the ABCD Study

The advantages of using the ABCD data set were (a) national sample, (b) large sample size, (c) large sample of NHWs and NHBs, (d) publicly available data, and (e) considerable socioeconomic, and behavioral variables [49,50,51,52,53].

### 2.3. Participants and Sampling

Participants of the ABCD study were selected across multiple cities across states. This sample was mostly recruited through school systems. A detailed description of the sampling of the ABCD is available here [55].

### 2.4. Study Variables

The study variables included demographic factors, SES indicators, and exposure to childhood trauma.

### 2.5. Independent Variable

*Parental Educational Attainment.* Participants were asked, “What is the highest grade or level of school you have completed or the highest degree you have received?” Responses were 0 = Never attended/Kindergarten only; 1 = 1st grade; 2 = 2nd grade; 3 = 3rd grade; 4 = 4th grade 4; 5 = 5th grade; 6 = 6th grade 6; 7 = 7th grade 7; 8 = 8th grade; 9 = 9th grade; 10 = 10th grade 10; 11 = 11th grade; 12 = 12th grade; 13 = high school graduate; 14 = GED or equivalent diploma; 15 = some college; 16 = associate degree: occupational; 17 = associate degree: academic program; 18 = bachelor’s degree (ex. BA; 19 = master’s degree (ex. MA; 20 = professional school degree (ex. MD; 21 = doctoral degree. This variable was an interval measure with a range between 1 and 21, with a higher score indicating higher educational attainment.

*Family income.* Family income was a continuous measure ranging from 1 to 10, with a higher score indicating higher income. The exact question was, “What is your total combined family income for the past 12 months? This should include income (before taxes and deductions) from all sources, wages, rent from properties, social security, disability and veteran’s benefits, unemployment benefits, workman”. Responses included 1 = Less than $5000; 2 = $5000; 3 = $12,000; 4 = $16,000; 5 = $25,000; 6 = $35,000; 7 = $50,000; 8 = $75,000; 9 = $100,000; 10 = $200,000.

### 2.6. Outcome

*Childhood Trauma.* Parents were interviewed regarding the trauma experienced by the child. The Kiddie Schedule for Affective Disorders and Schizophrenia (K-SADS) [56,57,58] was used to measure trauma. This is a semi-structured interview aimed at the early detection of high-risk youth. The items included: (1) “A car accident in which your child or another person in the car was hurt bad enough to require medical attention”, (2) “Another significant accident for which your child needed specialized and intensive medical treatment”, (3) “Witnessed or caught in a fire that caused significant property damage or personal injury”, (4) “Witnessed or caught in a natural disaster that caused significant property damage or personal injury”, (5) “Witnessed or present during an act of terrorism (e.g., Boston marathon bombing)”, (6) “Witnessed death or mass destruction in a war zone”, (7) “Witnessed someone shot or stabbed in the community”, (8) “Shot, stabbed, or beaten brutally by a non-family member”, (9) “Shot, stabbed, or beaten brutally by a grown up in the home”, (10) “Beaten to the point of having bruises by a grown up in the home”, (11) “A non-family member threatened to kill your child”, (12) “A family member threatened to kill your child”, (13) “Witness the grownups in the home push, shove or hit one another”, (14) “A grown up in the home touched your child in his or her privates, had your child touch their privates, or did other sexual things to your child”, (15) “An adult outside your family touched your child in his or her privates, had your child touch their privates or did other sexual things to your child”, (16) “A peer forced your child to do something sexually”, and (17) “Learned about the sudden unexpected death of a loved one”. Response items for each item were 0 (no) or 1 (yes). We counted the number of traumatic events, and, given the extreme skewness of the count of traumatic events, we calculated a variable as zero traumatic events, one traumatic event, and two or more traumatic events (Cronbach’s alpha = 0.637). Trauma research both in children and adults has frequently operationalized trauma as a three-level categorical variable: none, one, two, or more. This is because the consequences of trauma are intensity-dependent; thus, there is a need to collect data on the number of traumatic events. Multiple traumas (2+) are an indicator of a more stressful situation and have a stronger effect on a wide range of psychosocial outcomes [59,60,61,62,63,64,65,66,67].

### 2.7. Moderator

*Race/Ethnicity.* In the ABCD study, race was a self-identified variable with two levels, blacks, and whites (reference category). Ethnicity was also self-identified as a Hispanics and non-Hispanics (reference category). In the current analysis, race/ethnicity was a categorical variable: NHB = 1, NHW = 0.

### 2.8. Confounders

Age, parental marital status, and gender were the confounders. Parents reported the age of their children. Age was calculated as the difference between the date of birth to the date of enrolment to the study. Age was a continuous measure in years. Parental marital status, a dichotomous variable, was self-reported by the interviewed parent: married = 1 and other = 0. Given that gender is known to be correlated with exposure to childhood trauma (higher in girls than boys) [68], we used gender as a covariate (dichotomous variable: males = 1 and females = 0).

### 2.9. Data Analysis

We used the SPSS statistical package for our data analysis. Mean (standard deviation [SD]) and frequency (%) were reported for descriptive purposes. To perform our multivariable analyses, we performed four polynomial regression models. The first two models were performed in the pooled sample. Model 1 was performed without the interaction terms. Model 2 also included two interaction terms between race/ethnicity with parental educational attainment and family income. These models controlled for age, gender, and marital status. The outcome was exposure to childhood traumatic events. Predictors were parental educational attainment and family income. The next models were specific to race/ethnicity. Predictors were parental educational attainment and family income. The outcome was exposure to childhood trauma (0, 1, 2+). These models controlled for age, gender, and marital status. Unstandardized odds ratio (OR), 95% CI, and *p*-value were reported for each model.

### 2.10. Ethical Aspect

The ABCD study protocol was approved by the University of California, San Diego (UCSD) Institutional Review Board (IRB). All participants gave assent. Parents signed informed consent. More detailed information on ABCD study ethics is available elsewhere [54]. As we used fully de-identified data, our study was non-human subject research. Thus, it was exempted from a full review.

## 3. Results

### 3.1. Descriptive Data

Table 1 presents descriptive statistics of the pooled sample and by race/ethnicity. The current analysis was performed on 4696 8–11-year-old children who were either NHWs (n = 3321; %70.7) and NHBs (n = 1375; %29.3). NHB and NHW children did not differ in age and gender; however, they differed in parental marital status, parental education, and family income. NHB and NHW children also varied in exposure to childhood trauma.

### 3.2. Multivariate Analysis

Table 2 shows the results of two polynomial regression models in the overall (pooled) sample. Model 1 (Main Effect Model) showed protective effects of high parental education (OR = 0.96; 95% CI = 0.92–0.99) on exposure to one traumatic event and protective effects of family income (OR = 0.90; 95% CI = 0.85–0.95) on exposure to 2+ childhood traumatic events. In this model, NHWs reported a lower risk of 2+ traumatic events (OR = 0.76; 95% CI = 0.59–0.97), relative to NHBs. Model 2 (Interaction Model) showed two interaction terms between race/ethnicity with parental educational attainment and family income on exposure to childhood trauma, suggesting that the protective effect of family income against exposure to childhood trauma is weaker for NHB children relative to their NHW counterparts (Table 2).

### 3.3. Multivariate Analysis by Race/Ethnicity

Table 3 shows the results of Poisson regression models specific to NHWs (Model 3) and NHBs (Model 4). Model 3 showed the protective effects of parental education and family income against exposure to childhood traumatic events. Model 4 did not show any protective effects of parental education and family income on exposure to childhood trauma.

Table 3 shows *p* < 0.01 three times for education and income of NHW families (left column), but never for NHB families (right column). This suggests higher educational attainment and family income does not seem to reduce exposure to childhood trauma for NHB youth. However, the same resources reduce traumatic experiences for NHW adults. That means NHB children are going to experience a high level of childhood trauma at all family SES levels. To give an example, for NHW kids, an additional 10% more family income would mean a 24% reduction in the odds of exposure to multiple (2+) trauma. A similar 10% increase in family income would not reduce the odds of exposure to multiple traumas for NHB children. Similarly, a one-year increase in parental education would be associated with 8% and 9% decline in exposure to one and multiple traumas for NHW children, respectively. The same increase in parental education would not reduce exposure to one or multiple (2+) traumatic events for NHBs.

## 4. Discussion

Our findings showed that, although parental educational attainment and family income reduce exposure to childhood trauma, these protective effects are weaker for NHBs than NHWs. Thus, we observe diminishing returns of parental educational attainment and family income on exposure to childhood trauma for NHBs, relative to NHWs. That means high-SES NHB children experience higher than expected exposure to childhood trauma. High-SES NHW children, however, report a low level of childhood trauma.

Similar to our findings, MDRs are repeatedly shown for almost all SES resources, age groups, outcomes, and marginalization types [38,39]. The literature has documented MDRs of SES indicators, including family income and parental educational attainment for NHB compared to NHW families and individuals [40,43,69,70]. MDRs within families contribute to the trans-generational transmission of inequalities from one to another generation. The current paper suggests that disproportionately high risk of exposure to childhood trauma may explain why NHB children [40,43,69,70] and adults [30,31,71,72,73] still develop poor behavioral, physical, and mental health outcomes.

At all education and income levels, NHB families face disproportionately more stress in their daily lives [37]. Highly educated NHB are more likely to be surrounded by NHW families and attend predominantly-NHW schools and work in predominantly-NHW workplaces, all increasing their exposure to discrimination [32,33]. High levels of discrimination reduce the expected gains of SES [28,74,75]. High-SES NHB parents also experience a higher level of occupational stress and are exposed to worse environmental conditions, in part because they may work in worse jobs than NHWs with the same SES [34,35,43,76]. High-SES NHBs may also be more, rather than less vulnerable to interpersonal discrimination [75]. This increased stress may reduce how much parents and children can engage, thus limiting how much children can gain from their available parent education. High-income NHB families may experience more, not less discrimination on a daily basis [28,32,33,77,78].

Previous research has also shown that high-SES NHB students tend to attend schools that have high-risk peers [79]. This finding suggests that residential segregation may limit the school options for children from high-SES NHB families. As a result, children of high-SES NHB families continue to attend high-risk schools at highly segregated schools. Such schools have a higher risk environment across levels and domains from peers’ drug use, aggression, and behavioral problems. This pattern is different from high-SES NHW children who receive education at predominantly NHW schools that receive more funding and higher-quality teachers [80]. However, even for NHWs, marginalization reduces the return of SES [81].

As suggested by the MDRs theory [38,39], equal SES resources result in unequal outcomes, with marginalized and stigmatized social groups experiencing a relative disadvantage in comparison to the socially privileged group. MDRs seem to be robust across age groups, SES resources, and outcomes. More than other SES indicators, however, educational attainment has shown differential effects by racial and ethnic groups, with NHBs benefitting less than NHWs. This occurs not only with income [44] but also educational attainment [40], employment [82], income [83], and marital status [45]. Family SES results in greater gains for NHW than NHB children [44,47,48], adults [43], and older adults [84]. Additionally, MDRs not only apply to NHBs [48], as they also hold for Asian Americans [85], Native Americans [86], Hispanics [40,87,88,89], and LGBTQs [69].

The current study did not explore societal and contextual processes that could explain intergenerational MDRs of education for racial and ethnic minorities. MDRs may be due to institutional and structural racism [38,90]. Marginal returns may also be smaller for families with a history of childhood poverty [91]. Racial prejudice and discrimination may also interfere with the benefit that is expected to follow education [31,72,73]. Thus, multilevel economic, psychological, and societal mechanisms may be involved in explaining racial and ethnic gaps in the returns of parental education [38,90].

One hypothesis is that NHB individuals are likely to stay in poor neighborhoods despite high SES [79,81]. As a result, children from high-SES NHB families may remain at risk of environmental exposure to risk factors. In such a context, high risk of peer discrimination/aggression and poor school quality may reduce the effect of family SES for NHB families [85,92]. However, more research is needed on the role of peers and neighborhoods and schools in explaining the high risk of high-SES NHB children and youth. We already know that being in high-risk neighborhoods and interacting with high-risk peers are major risk factors for behavioral problems and poor outcomes [93]. As the environment is modifiable by policy, there is a need to address segregation and environmental risk in predominantly NHB schools and neighborhoods [94].

The society and social structure may be critical conduits through which MDRs are developed and sustained. As a result, additional attention should be paid to various societal processes that may interfere with the returns of educational outcomes. According to the social reproduction theory, intergenerational educational outcomes may vary across groups [95]. Chetty showed that the intersection of race and gender alters the likelihood of upward social mobility in the US [96].

### Limitations

No study is without methodological limitations. The main limitations of this study include the cross-sectional design. Similar to most of the literature on MDRs, this study focused exclusively on NHBs and NHWs. We still need more studies on other ethnic groups such as Asian Americans and Native Americans as well as other marginalizing identities beyond race and ethnicity. Not only race and ethnicity, but all marginalizing identities potentially reduce the gains that follow educational attainment [69,88,89,97]. Similarly, this study only investigated the MDRs of parental education and family income. Other research may study other SES indicators such as wealth, and higher-level SES indicators such as neighborhood SES and income or racial segregation similarly influence outcomes across race/ethnic groups, or such contextual factors can explain MDRs. Future research may study structural and contextual factors that explain the MDRs of family SES.

Although some critics may argue that the breaking down of childhood trauma into “1 traumatic event” and “2+ traumatic events” is somewhat arbitrary, this was our strategy because of the skewed distribution of childhood trauma; most children did not report any trauma, and a very small proportion of families reported more than three traumas. While we were limited by the distribution of our data (variable), we wanted to test if MDRs are relevant to any trauma or higher dosage of the trauma. By operationalizing our outcome as a three-level categorical variable, we observed that family SES might be similarly protective against a low level of childhood trauma, but SES is far more protective for NHW than NHB families in reducing exposure to 2+ childhood traumas. Thus, we could observe that MDRs are observed because some NHB families with high SES are still exposed to multiple traumas, a pattern which is absent in NHWs, given the high protective effect of SES for them.

## 5. Conclusions

Compared to NHWs, NHB children from high-SES families remain at high risk of exposure to childhood trauma, particularly two or more traumatic events. Social stratification, racism, segregation, prejudice, and discrimination may be responsible for why NHBs from high-SES families remain at high risk of exposure to childhood trauma. High exposure to childhood trauma may also explain why high-SES NHB children youth and adults develop worse-than-expected behavioral, mental, and physical health outcomes.

## Figures and Tables

**Table 1:** Socio-demographic data overall and by race/ethnicity **(*n* =** **4696)**.

	All		NHWs		NHBs	
	*n*	%	*n*	%	*n*	%
Race/Ethnicity						
NHWs	3321	70.7	3321	100.0	-	-
NHBs	1375	29.3	-	-	1375	100.0
Gender						
Male	2236	47.6	1569	47.2	667	48.5
Female	2460	52.4	1752	52.8	708	51.5
Marital Status *						
Other	1255	26.9	435	13.1	820	60.8
Married	3415	73.1	2886	86.9	529	39.2
Exposure to Childhood Trauma *						
0	2953	62.9	2207	66.5	746	54.3
1	1251	26.6	865	26.0	386	28.1
2+	492	10.5	249	7.5	243	17.7
	**Mean**	**SD**	**Mean**	**SD**	**Mean**	**SD**
Age (Year)	9.44	0.50	9.44	0.50	9.45	0.52
Parental Educational Attainment (0–21) *	16.92	2.38	17.59	1.99	15.33	2.51
Family Income (1–10) *	7.25	2.50	8.16	1.73	5.04	2.69

Standard deviation (SD). Non-Hispanic white (NHW). Non-Hispanic black (NHB). * *p* < 0.05 for comparison of NHBs and NHWs.

**Table 2:** Summary of polynomial regressions overall **(*n* = 4696)**.

	Model 1 (Main Effects)			Model 2 (Interactions)		
	OR	95% CI	*p*	OR	95% CI	*p*
Outcome: 1 Trauma						
Race (NHW)	0.94	0.78–1.13	0.518	13.13	4.47–38.57	<0.001
Gender (Female)	1.12	0.98–1.28	0.091	1.14	0.99–1.30	0.063
Marital Status (Non-Married)	1.41	1.17–1.69	<0.001	1.42	1.18–1.71	<0.001
Age	1.07	0.94–1.22	0.331	1.06	0.93–1.21	0.391
Parental Educational Attainment	0.96	0.92–0.99	0.017	0.92	0.88–0.96	<0.001
Family Income	1.02	0.98–1.07	0.352	0.95	0.90–1.01	0.110
Parental Educational Attainment × NHB				1.12	1.04–1.21	0.003
Family Income × NHB				1.12	1.04–1.21	0.005
Intercept			0.162			0.003
Outcome: 2+ Trauma						
Race (NHW)	0.76	0.59–0.97	0.030	53.10	12.79–220.51	<0.001
Gender (Female)	1.00	0.82–1.22	0.988	1.03	0.85–1.26	0.745
Marital Status (Non-Married)	1.99	1.53–2.58	<0.001	1.84	1.43–2.38	<0.001
Age	1.02	0.84–1.23	0.863	0.99	0.81–1.20	0.918
Parental Educational Attainment	0.98	0.93–1.03	0.352	0.91	0.85–0.98	0.014
Family Income	0.90	0.85–0.95	<0.001	0.75	0.70–0.81	<0.001
Parental Educational Attainment × NHB				1.16	1.05–1.29	0.004
Family Income × NHB				1.34	1.22–1.48	<0.001
Intercept			0.368			0.023

CI = Confidence interval. Non-Hispanic white (NHW). Non-Hispanic black (NHB).

**Table 3:** Summary of polynomial regressions by race/ethnicity **(*n* = 4696)**.

	Model 3 (NHWs)			Model 4 (NHBs)		
	OR	95% CI	*p*	OR	95% CI	*p*
Outcome: 1 Trauma						
Gender (Female)	1.18	1.00–1.38	0.046	1.04	0.81–1.33	0.749
Marital Status (Non-Married)	1.60	1.27–2.02	<0.001	1.13	0.83–1.54	0.436
Age	1.07	0.91–1.25	0.439	1.04	0.82–1.33	0.727
Parental Educational Attainment	0.92	0.88–0.96	<0.001	1.03	0.97–1.09	0.353
Family Income	0.97	0.91–1.02	0.246	1.05	0.98–1.12	0.153
Intercept			0.910			0.140
Outcome: 2+ Trauma						
Gender (Female)	1.01	0.77–1.32	0.971	1.05	0.78–1.40	0.752
Marital Status (Non-Married)	1.92	1.37–2.70	<0.001	1.71	1.16–2.51	0.007
Age (Years)	0.94	0.71–1.23	0.628	1.05	0.79–1.39	0.753
Parental Educational Attainment (1–21)	0.91	0.85–0.98	0.013	1.06	0.99–1.14	0.112
Family Income (1–10)	0.76	0.70–0.82	<0.001	1.01	0.93–1.08	0.894
Intercept			0.157			0.051

Odds ratio (OR). Confidence interval (CI). non-Hispanic white (NHW). non-Hispanic black (NHB).
